# Undifferentiated Embryonal Sarcoma of the Liver in an Adult Patient

**DOI:** 10.7759/cureus.3037

**Published:** 2018-07-24

**Authors:** Kemal Beksac, Rustam Mammadov, Turkmen Ciftci, Gunes Guner, Aytekin Akyol, Volkan Kaynaroglu

**Affiliations:** 1 General Surgery, Dr. Abdurrahman Yurtarslan Ankara Oncology Education and Research Hospital, Ankara, TUR; 2 Surgery, Hacettepe University, Ankara, TUR; 3 Interventional Radiology, Hacettepe University, Ankara, TUR; 4 Pathology, Hacettepe University, Ankara, TUR; 5 General Surgery, Hacettepe University, Ankara, TUR

**Keywords:** undifferentiated embryonal sarcoma of the liver, hepatic malignancy, hepatic resection, percutaneous transhepatic cholangiography

## Abstract

An undifferentiated embryonal sarcoma of the liver (UESL) is a rare and highly malignant mesenchymal neoplasm that is uncommonly observed in adults. We report a case of UESL found in a 26-year-old female. Our case was initially regarded as a type II hydatid cyst and then a malignant mass in radiological studies. The patient underwent nonanatomic liver resection. There were postoperative complications, but they were handled successfully. The patient received taxol-cisplatin-ifosfamide chemotherapy protocol and is disease-free after six years. Although UESL is exceedingly rare in adults, it must be considered while evaluating large hepatic masses since curative resection has an excellent prognosis.

## Introduction

An undifferentiated embryonal sarcoma of the liver (UESL), also called a malignant mesenchymoma of the liver, is a rare and highly malignant mesenchymal neoplasm that was first described in 1978 [1]. A study in 2016 reports only 198 patients in 23 series [2]. It is primarily a disease of pediatric patients and is composed of poorly differentiated cells of undetermined origin other than mesenchymal. It is a unique sarcoma of the liver due to specific clinical and histologic characteristics that no other soft tissue tumors have [3]. It was originally regarded as a very aggressive tumor with a median survival of less than one year, but due to advanced liver resections, supportive treatment modalities, and chemotherapeutic agents, many patients can be cured at present [1,4]. Surgery is mandatory in these patients due to plausible complications, such as intratumoral bleeding and rupture, which lead to high mortality [5-6]. Here, we report a UESL case in an adult patient and review the relevant literature.

## Case presentation

**Figure 1:**
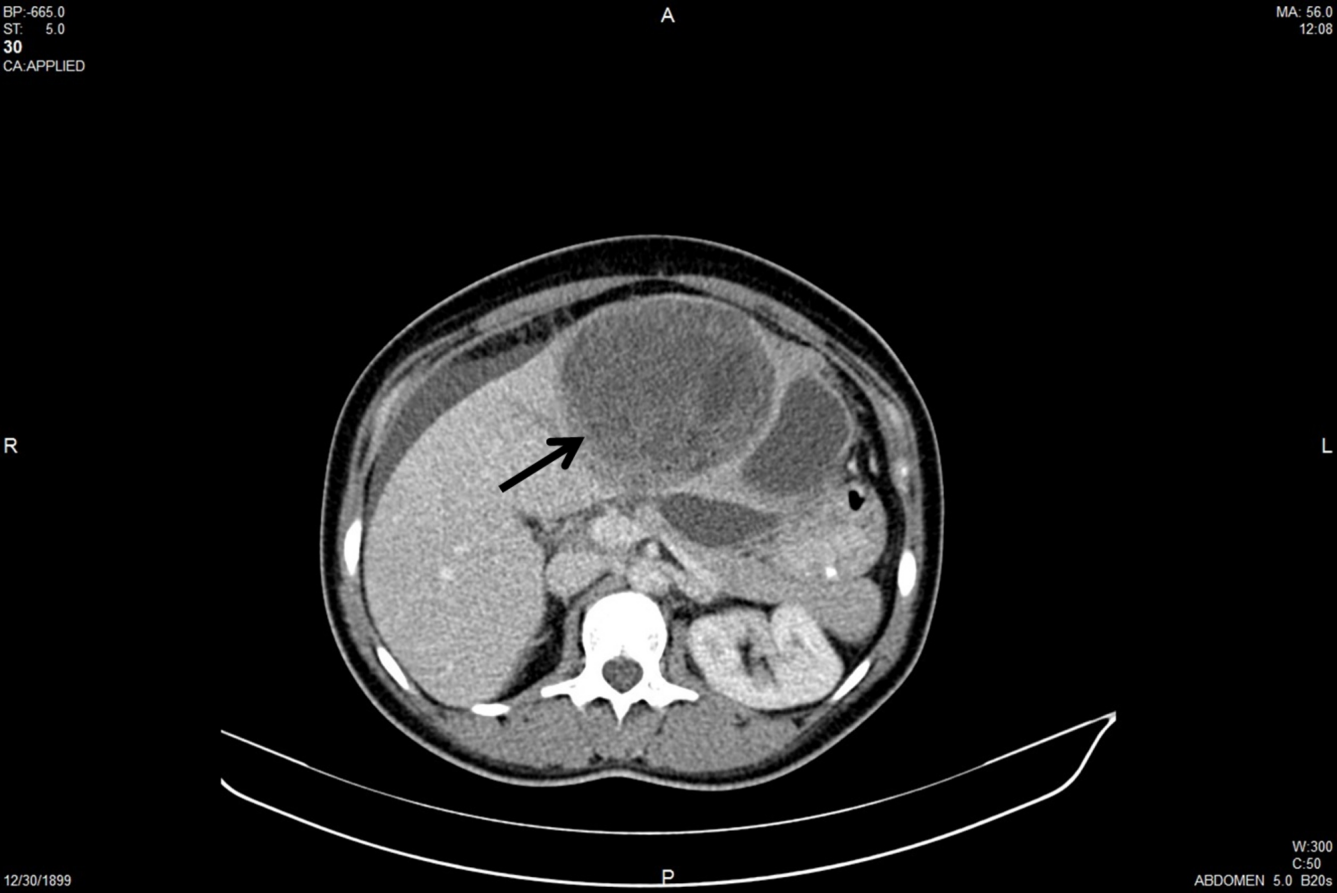
Preoperative contrast-enhanced computerized tomography image of the mass

A 26-year-old female patient was admitted for a one-month-old abdominal pain in February 2012. The initial evaluation was consistent with a 10-cm type II hydatid cyst in the liver, and she was, therefore, referred to our center. Abdominal computed tomography (CT) in our center revealed a 17x12x17 cm mass with solid and cystic components in the left lobe of the liver (Figure 1).

The tumor size and the location and cystic nature of the mass suggested biliary cystadenocarcinoma as a possible diagnosis. A biopsy of the mass showed an inflamed myxoid stroma with necrosis and fine needle aspiration cytology of the cystic fluid revealed necrosis with minimally atypical glandular epithelial cells.

The patient’s physical evaluation was normal, except for the palpable mass in the epigastric region. Laboratory findings, liver function tests, and tumor markers (CA 19-9, CEA, and CA-125) were all normal.

The patient's surgical procedures and outcomes were as follows: nonanatomic liver, cholecystectomy, and extrahepatic biliary tract resection were performed. The right lobe anterior segment hepatic duct was anastomosed to the jejunum in Roux-en-Y fashion. The right lobe posterior segment hepatic duct was anastomosed to the common bile duct.

**Figure 2:**
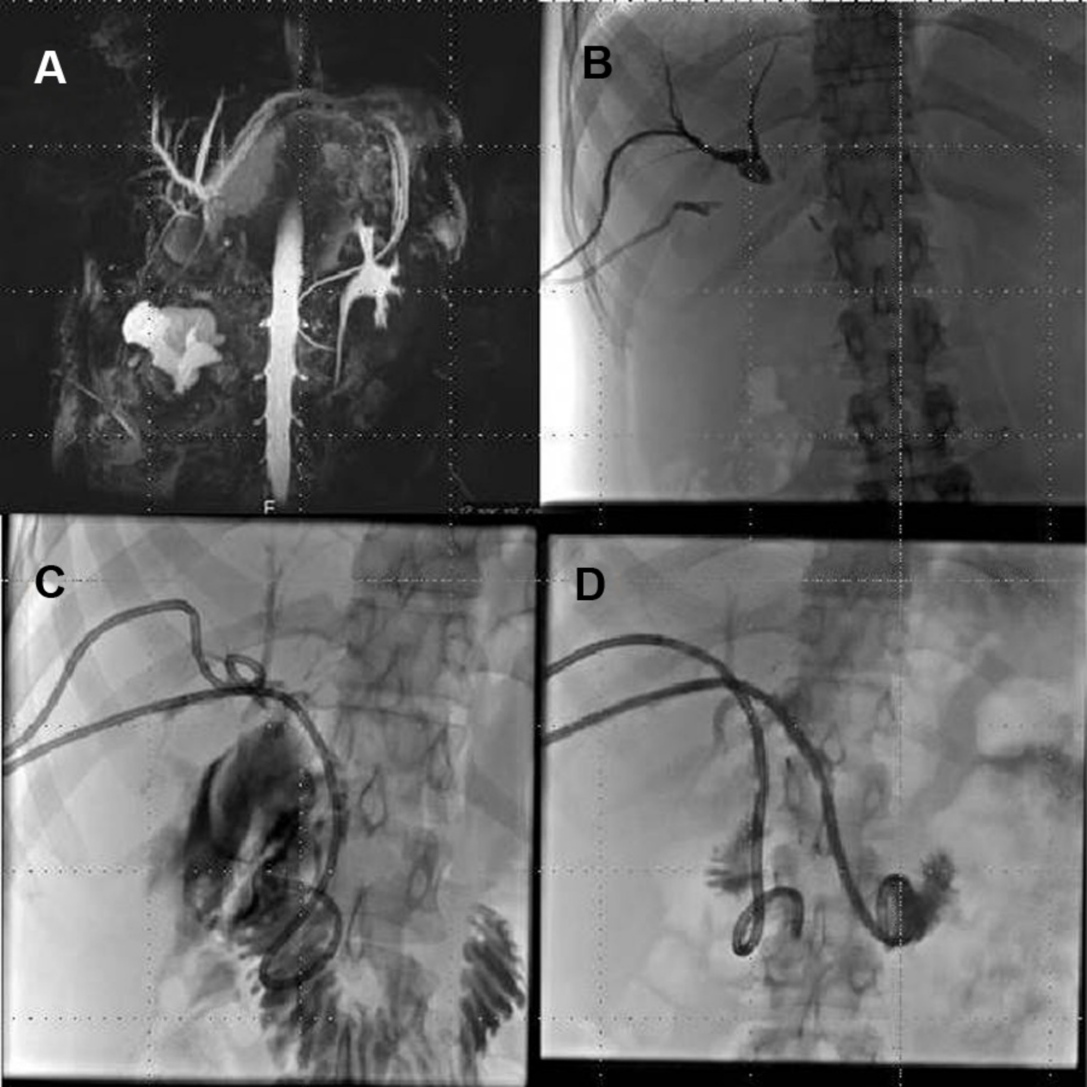
A. Intrahepatic bile duct dilation secondary to obstruction. B. Placement of external biliary drainage catheter for each anastomosis. C. Placement of an internal-external biliary drainage catheter through hepatojejunostomy. D. Placement of the internal-external drainage catheter through the newly created anastomosis.

Bile leakage developed after the operation. Postoperative magnetic resonance imaging and magnetic resonance cholangiopancreatography revealed a leakage from the hepaticojejunostomy. Furthermore, intrahepatic bile duct dilatation was observed secondary to stenosis in both anastomoses (Figure 2a). The patient was referred to the interventional radiology unit. First, the infected bile collection was drained under ultrasonography and fluoroscopy guidance. Following the resolution of the collection, percutaneous transhepatic cholangiography (PTC) was performed. PTC revealed that the leakage was healed. Eight FR external biliary drainage catheters were placed for each anastomosis separately (Figure 2b). Next, a guide wire was inserted through the stenosis at the hepaticojejunostomy site and balloon dilatation was performed. An internal-external biliary drainage catheter was placed in this position (Figure 2c). However, stenosis located at the anastomosis between the right lobe posterior hepatic duct and the common bile duct could not be managed percutaneously. Therefore, open surgery was planned. Under intraoperative fluoroscopy, a guide wire was directed through the patients’ external biliary drainage catheter. This wire was guided to puncture the biliary tract proximally from the stenosis and through the liver parenchyma into the abdomen. The blind loop of jejunum was moved toward the visible catheter and a mucosal graft was applied on the catheter through a small opening made on the jejunum segment. A 10 FR internal-external biliary drainage catheter was placed at the site of this anastomosis (Figure 2d).

**Figure 3:**
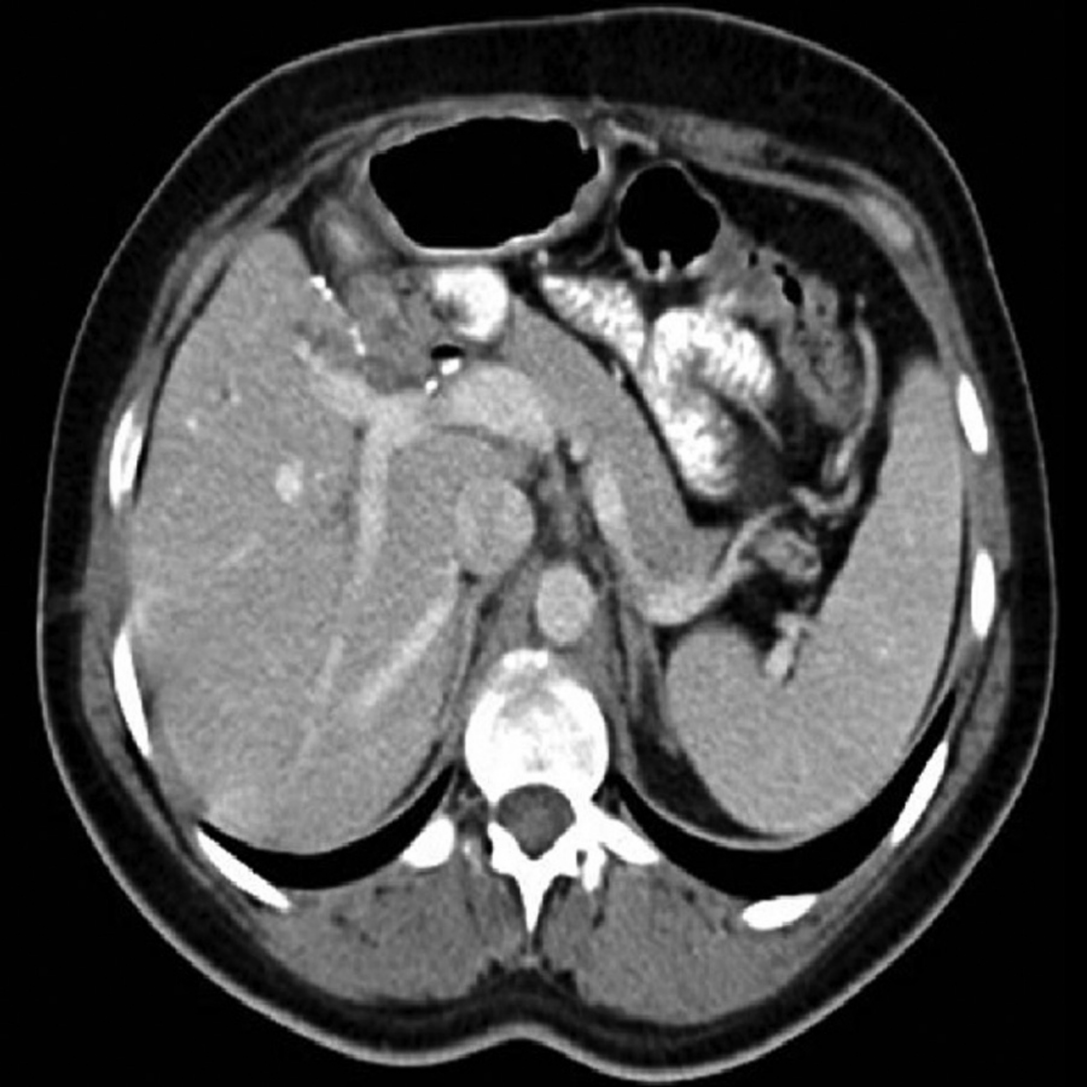
Postoperative contrast-enhanced computerized tomography image.

A single drain was placed near the newly created anastomosis. This time, the postoperative period was uneventful, and the patient was eventually discharged successfully. Three months later, control cholangiography revealed that both of the anastomoses were healed perfectly. Both biliary drainage catheters were consequently removed. A postoperative contrast-enhanced CT image is presented in Figure 3.

**Figure 4:**
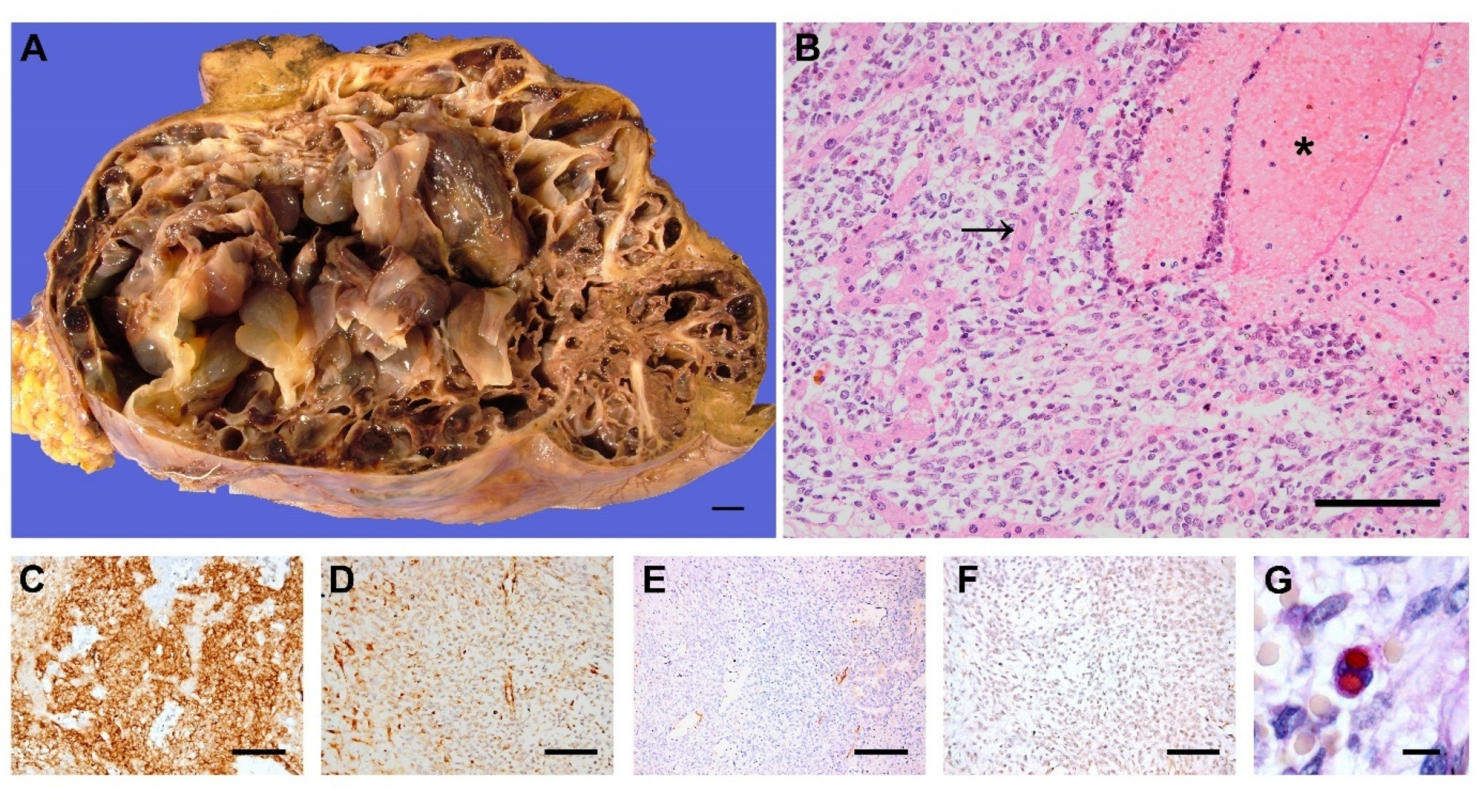
A. The tumor indicated was 25 cm in its largest dimension and consisted of multiple areas of cystic degeneration separated by gray to tan septae of variable thickness (scale bar, 1 cm). B. Non-neoplastic hepatocyte cords (arrow) were infiltrated by spindle to oval-shaped, pleomorphic neoplastic cells. Foci of hemorrhage were common (asterisk) (H-E, x20, scale bar 100 um). Neoplastic cells were diffusely positive for CD56 (C), focally positive for vimentin (D) and desmin (E). Pancytokeratin was negative (F). PAS-positive, diastase-resistant intracytoplasmic material was detected in a rare neoplastic cell (G) (C-F, x20, scale bar 100 um. G, x40, scale bar 5 um).

The excision specimen consisted of a 25x19x10 cm well-circumscribed mass with small remnants of non-neoplastic, non-cirrhotic hepatic parenchyma at the periphery. The lesion was gray-tan in color and was made up of multiple areas of cystic degeneration, fragile fibrous septae, areas of hemorrhage, and myxoid change (Figure 4a). Necrosis was not prominent on a macroscopic examination. A microscopic examination revealed widespread cystic degeneration, hemorrhage, and necrosis along with small hypercellular nodules and myxoid areas harboring neoplastic cells. Neoplastic cells displayed moderate pleomorphism and nuclear hyperchromasia. Although the lesion seemed well-circumscribed macroscopically, microscopy showed tumor cells infiltrating between hepatocyte cords (Figure 4b). Immunohistochemically, neoplastic cells were strongly and diffusely positive for CD56 (Figure 4c) and focally positive for vimentin (Figure 4d), CD10, WT1 (cytoplasmic), Factor VIII, and desmin (Figure 4e). Pancytokeratin (CK) (Figure 4f), ER, PR, CD31, lysozyme, smooth muscle actin, CD68, myoD1, HepPar1, and CD34 were negative in tumor cells. Rare PAS-positive, diastase-resistant, intracytoplasmic material was detected in some neoplastic cells (Figure 4g).

The proliferation index (PI), as demonstrated by Ki67 immunohistochemistry, was approximately 30% (not shown). In some cells that lined the multicystic-degenerated areas, rare intracytoplasmic mucin droplets were noted. These cells expressed CK19 and were considered to be remnants of trapped bile duct elements (not shown).

After surgery, the patient received the taxol-cisplatin-ifosfamide chemotherapy protocol (Table 1). The patient is currently disease-free after six years of follow up.

**Table 1:** Taxol-Cisplatin-Ifosfamide chemotherapy protocol i.v.: intravenous, s.c.: subcutaneous, sqm: square meter

Chemotherapeutic	Dosage	Administration Method	Application Days
Paclitaxel	175 mg/sqm	i.v.	Day 1
Cisplatin	20 mg/sqm	i.v.	Day 1-5
Ifosfamide	1000 mg/sqm	i.v.	Day 1-5
Mesna	1000 mg/sqm	i.v.	Day 1-5
Pegfilgastrim	6 mg	s.c.	Day 6

## Discussion

UESL is a rare primary mesenchymal tumor in children but there have been a few reports in adults as well [7-10]. These often present as masses with solid and cystic components. Due to the rarity of UESL in adult patients, these patients are often misdiagnosed as hepatic abscess, hemorrhage cystic tumor, or hydatic cyst, as in this case [11].

UESL has no specific clinical characteristics. Some patients may present with various nonspecific gastrointestinal symptoms, such as nausea, vomiting, abdominal pain, diarrhea, and jaundice. A large liver mass along with persistent weight loss is apparent in most adult cases. UESL is not associated with cirrhosis. Liver functions and tumor markers, such as AFP, CEA, and CA 19-9, are normal in most cases, such as this one. UESL can be observed in either of the lobes of the liver and sometimes in both of them [12-13].

Macroscopically, UESL is a large, well-circumscribed mass with areas of cystic degeneration, necrosis, hemorrhage, and occasional gelatinous appearance, and the tumor is surrounded by a fibrous pseudocapsule with the direct invasion of the adjacent parenchyma, consistent with our findings. The cellular component is composed of medium to large spindled or stellate cells with marked nuclear pleomorphism [14-15]. Although its pathologic origin is unclear, studies have shown histiocytic, lipoblastic, myoblastic, myofibroblastic, rhabdomyoblastic, and leiomyoblastic differentiation [16]. UESLs are usually diffusely positive for vimentin and a1-AT and focally positive for cytokeratin, desmin, α-SMA, muscle-specific actin, CD68, myoglobin, neuron-specific enolase, S100, and CD34, suggesting that an embryonal sarcoma is undifferentiated [17]. Diffuse CD56 immunoreactivity was reported in eight UESL cases and is an emerging biomarker to help the diagnosis of UESL [18]. Periodic Acid Schiff positive and diastase-resistant intra- and extracytoplasmic globules may be observed in UESL [18], as was seen in our case. Cytology material contained sloughed-off biliary epithelial cells of preexisting bile ductules trapped between the degenerated masses and fibrous septae of the tumor.

The standard treatment of UESL includes complete resection of tumor and combined adjuvant chemotherapy [19]. There is no universally accepted chemotherapy protocol for UESL. In our case, we utilized the taxol-cisplatin-ifosfamide protocol indicated in Table 1. Liver transplantation may also be an option for patients with unresectable tumors. Radical resection is also recommended for recurrent cases [4]. While the initial reports gave unfavorable results, advances in liver resection techniques and supportive modalities improve outcomes, as demonstrated in our study. This patient had a troubled postoperative period due to stenosis in anastomoses. However, they were managed successfully due to modern techniques, particularly those related to interventional radiology. Nowadays, long-term survival rates are reported to be between 70% and 100% [20]. This patient is disease-free after six years of follow up.

## Conclusions

UESL is a challenging diagnosis. It should be included in the differential diagnoses of large liver masses, regardless of patient age. It is an aggressive tumor, but a combined therapy of complete resection and chemotherapy has a very good prognosis. Complete resection is the most important step for long-term survival.
